# Removing recalcitrance to the micropropagation of five farmer-preferred cassava varieties in Côte d’Ivoire by supplementing culture medium with kinetin or thidiazuron

**DOI:** 10.3389/fpls.2025.1538799

**Published:** 2025-04-15

**Authors:** John Steven S. Seka, Modeste K. Kouassi, Edwige F. Yéo, Flavie M. Saki, Daniel H. Otron, Fidèle Tiendrébéogo, Angela Eni, Nazaire K. Kouassi, Justin S. Pita

**Affiliations:** ^1^ Unité de Formation et de Recherche Biosciences, Université Félix Houphouët-Boigny (UFHB), Abidjan, Côte d’Ivoire; ^2^ The Central and West African Virus Epidemiology (WAVE) for Food Security Program, Pôle Scientifique et d’Innovation, Université Félix Houphouët-Boigny (UFHB), Abidjan, Côte d’Ivoire; ^3^ Unité de Formation et de Recherche (UFR) d’Ingénierie Agronomique forestière et Environnementale, Université de Man (UMAN), Man, Côte d’Ivoire

**Keywords:** *in vitro* propagation, recalcitrant cassava varieties, thidiazuron, kinetin, virus-free plantlets

## Abstract

*In vitro* micropropagation is a rapid method of multiplying healthy planting material to control Cassava mosaic disease (CMD), one of a major constraint to cassava production in Africa. However, some cassava varieties have a low propagation ratio under *in vitro* conditions. The main objective of this study was to improve the *in vitro* propagation rate of five difficult to grow, farmer-preferred cassava varieties using plant growth regulators. Microcuttings from *in vitro* plantlets of five recalcitrant cassava varieties (Agbablé 3, Ampong, Bayérè, Bocou 5, Olékanga) were evaluated for their capacity to rapidly regenerate plantlets. Time to root or leaf formation, number of nodes, number of roots, and the *in vitro* plantlet length were evaluated on nine culture media combinations. We found that among all the cassava varieties studied, the shortest times for leaf (4 to 7 days) or root (9 to 14 days) formation were recorded when the two types of MS media were supplemented with kinetin and thidiazuron as well as on the medium contain half-strength MS without these plant growth regulators. These two hormones evaluated were better for regeneration of leaves, nodes and elongation of *in vitro* plantlets with optimum concentration of 5 and 10 nM or thidiazuron, and 0.12 or 0.24 µM for KIN. A survival rate between 85-91% was recorded under tunnel conditions and the plantlets appeared to be morphologically normal. The information obtained during this study will be useful for mass multiplication programs of elite cassava varieties.

## 1 Introduction

Cassava (*Manihot esculenta* Crantz) belong to *Euphorbiaceae* family, which genus *Manihot* includes over 200 species. The most important is *M. esculenta* which is the main carbohydrate source for more than 800 million people in the world and particularly in tropical regions in Africa, Asia and Latin America (Rosenthal and Ort, 2012). In Côte d’Ivoire, cassava is the second most important food crop after yam with an annual production of more than 6.3 million tons in 2022 (FAOSTAT, 2022). However, cassava productivity is challenged by several biotic and abiotic constraints resulting in low yield. Among the biotic constraints, the most damaging is Cassava Mosaic Disease (CMD), which is responsible for yield losses between 50% and 70% (Chikoti et al., 2019). The CMD is endemic in Africa and is caused by nine begomovirus species “https://ictv.global/report/chapter/geminiviridae/geminiviridae/begomovirus (accessed 27 November 2024)”. The most effective way to control this disease is to use virus-free planting material.

Cassava is currently propagated vegetatively using cuttings, but the maximum cutting production of this conventional method is low at 1:10 rate (Vernier et al., 2018). Consequently, significant number of plants are required to obtain sufficient quantities of planting material. In addition to the low multiplication rate achieved with traditional vegetative propagation method, this method also promotes CMD propagation in the field (Kouakou et al., 2024).

The *in vitro* propagation technique makes it possible to sanitize several susceptible cassava varieties using thermotherapy combined with meristem culture. This is followed by mass multiplication of the disease-free material to make it available to farmers (Yéo et al., 2020; Nakabonge et al., 2020; Zango et al., 2021). The MS medium has therefore been used for the *in vitro* propagation of several plant species, with many advantages, particularly for species that are difficult to propagate vegetatively or by seed such as cassava. This mineral salts rich medium was developed by Murashige & Skoog in 1962 as a result of their work on tobacco soft callus (Murashige and Skoog, 1962). However, some varieties are difficult to grow on this common culture medium (MS), as they often require special growth factors or growth regulators and specific media compositions (Costa et al., 2019; Sesay et al., 2018; Apio et al., 2021).

*In vitro* propagation is regulated by endogenous hormones. Auxin is the main hormone that induces lateral budding and rooting (Pasternak and Steinmacher, 2024). The addition of exogenous hormones modulates the action of these endogenous hormones. However, the combined action of these hormones can be optimized by the addition of specific nutrients. Generally, exogenous plant growth regulators (PGRs) which include cytokinin such as 6-benzylaminopurine (BAP), kinetin (KIN) and thidiazuron (TDZ), and auxins such as α-naphthalene acetic acid (NAA) are combined with the MS medium to optimize cassava *in vitro* propagation (Sessou et al., 2020; Feyisa, 2021; Hamdeni et al., 2022). The type and quantity of PGRs added to the growth media depend on several factors, the most important being the plant genotype; therefore, it is necessary to optimize the concentrations of these growth regulators given their essential role in morphogenesis (Purohit et al., 2011; Okello et al., 2021).

The aim of this study is to develop an efficient *in vitro* mass propagation protocol for five *in vitro* difficult-to-grow cassava varieties to meet the needs of cassava farmers.

## 2 Materials and methods

### 2.1 Plant material preparation

*In vitro* plantlets of five commonly cultivated cassava varieties (Agbablé 3, Ampong, Bayérè, Bocou 5, Olékanga) which have been previously sanitized by thermotherapy and meristem culture were selected for this study. To check for the virus-free status of the plantlets, the presence or absence of the target viruses was determined by PCR using specific primers. These two process were carried out as described by Yéo et al. (2020).

### 2.2 Media preparation and culture conditions

#### 2.2.1 Media to identify the best type and concentration of cytokinin

To identify the best type and concentration, two cytokinin, such as kinetin (KIN) and thidiazuron (TDZ) at different concentrations were added on the commonly used full-strength Murashige and Skoog (1962) and the results were subsequently evaluated (**Table 1**). Briefly, 0.12, 0.24 and 0.36 μM KIN (Sigma) or 5 and 10 nM of TDZ (Sigma) were added separately to the full-strength MS media (Duchefa). The control was full-strength MS medium supplemented with BAP (0.22 µM) (Duchefa) according to the methods of Ng (1990) and Mapayi et al. (2013). These media were supplemented with 30 g/l sucrose (Sucaf), 100 mg/l myo-inositol (Duchefa), 0.05 µM α-naphthalene acetic acid (NAA) (Duchefa), and 7 g/L agar (Duchefa) before being adjusted to pH 5.7 and sterilized at 121°C for 20 minutes in 80 ml glass jars (8 cm diameter and 15 cm long).

**Table 1:** Formulation of the media evaluated for the cassava *in vitro* growing.

Media	MS medium concentration	Plant Growth Regulators			
		NAA (µM)	BAP (µM)	KIN (µM)	TDZ (nM)
M1 (control)	full-strength MS	0.05	0.22	–	–
M2	full-strength MS	0.05	–	–	5
M3	full-strength MS	0.05	–	–	10
M4	full-strength MS	0.05	–	0.12	–
M5	full-strength MS	0.05	–	0.24	–
M6	full-strength MS	0.05	–	0.36	–
M7	half-strength MS	0.05	0.22	–	–
M8	half-strength MS	0.05	–	0.24	–
M9	half-strength MS	0.05	–	–	10

MS, Murashige & Skoog medium; NAA, α-naphthalene acetic acid; BAP, 6-benzylaminopurine;

KIN, Kinetin; TDZ, Thidiazuron.

#### 2.2.2 Media to determine the best MS basal salt concentration

In order to develop a cost-effective protocol, two media with full-strength MS (MS) in one hand and half-strength MS (1/2 MS) in other hand were compared (**Table 1**). To obtain the half-strength MS, the quantities of macroelements, microelements and vitamins in the full-strength MS medium were reduced by half. Both media were supplemented with BAP (0.22 µM) (Duchefa) and 0.05 µM α-naphthalene acetic acid (NAA) (Duchefa) according to the methods of Ng (1990) and Mapayi et al. (2013). These media were supplemented with 30 g/l sucrose (Sucaf), 100 mg/l myo-inositol (Duchefa) and 7 g/L agar (Duchefa) before being adjusted to pH 5.7 and sterilized at 121°C for 20 minutes in 80 ml glass jars (8 cm diameter and 15 cm long).

#### 2.2.3 Media for the effect of the cytokinin concentrations on medium with the best strength of MS basal salt

To establish an efficient protocol, the best medium with the MS basal salt was tested with the best concentration of KIN on one side and the best concentration of TDZ on the other side (**Table 1**). These media were compared with the basal medium supplemented with 0.22 µM of 6-benzylaminopurine (BAP) (Duchefa) according to the methods of Ng (1990) and Mapayi et al. (2013). These media used were supplemented with 30 g/l sucrose (Sucaf), 100 mg/l myo-inositol (Duchefa), 0.05 µM α-naphthalene acetic acid (NAA) (Duchefa), and 7 g/L agar (Duchefa) before being adjusted to pH 5.7 and sterilized at 121°C for 20 minutes in 80 ml glass jars (8 cm diameter and 15 cm long).

### 2.3 Explants cultivation, growth room conditions and data collection

Some micro-cuttings with one node were taken from the three-month-old *in vitro* plantlets of the five cassava varieties. Five micro-cuttings of each variety were transplanted into a glass jar and the experiments were repeated six times. The culture was maintained in a growth room with a relative humidity of 85% and a temperature of 25°C ± 2°C. A photoperiod of 16 hours of light and 8 hours of dark was applied in the growth room using light-emitting diode (LED) lamps. For each explant, the time to rooting (rhizogenesis) and leaf formation (phyllogenesis) was assessed by counting the number of days from start of cultivation until the first root or leaf emerged. The average time (T) per jar was then calculated using Formula 1. This helped to determine which factors promoted the *in vitro* regeneration of the explant. In addition, the number of nodes, roots and the length of plantlets were also evaluated eight weeks after transplanting to determine which media formulation resulted in the most efficient organogenesis for each variety. The mean values of these parameters were calculated using Formulas 2-4.


(1) $$T=\frac{\sum time\;from\;transplanting\;until\;the\;emergence\;of\;first\;leaves\;or\;roots}{Total\;number\;of\;explants}$$

(2) $$Mean\;number\;of\;leaves=\frac{\sum number\;of\;leaves}{Total\;number\;of\;explants}$$

(3) $$Mean\;number\;of\;roots=\frac{\sum number\;of\;roots}{Total\;number\;of\;explants}$$

(4) $$Mean\;of\;in\;vitro\;plantlet\;length=\frac{\sum length\;of\;plantlet}{Total\;number\;of\;explants}$$

### 2.4 Acclimatization of *in vitro* plantlets

Regenerated *in vitro* plantlets with at least one well-developed root were carefully removed from the culture vessel, washed with water to remove the agar gel, and then transferred to plastic pots containing a substrate composed of soil and *Cocos nucifera* (L.) peat in a 2:1 ratio. Acclimatization was carried out in a tunnel with a relative humidity of 90% and a temperature between 30 and 40°C. After two weeks, the tunnels were opened to reduce the humidity and the plants were sprayed with water three times per week. Plant survival rate was recorded after four weeks and for up to eight weeks, after which surviving plantlets were removed from the tunnel.

### 2.5 Statistical analyses

A randomized design was used with at least six biological replicates per factor and each biological replicate was a glass-jar containing five explants from five different varieties. A total of 1350 explants were examined in our experiment (270 explants per variety). To determine the most appropriate statistical approach, the assumptions for performing parametric statistics were tested as follows; independence of observations was assessed using the Durbin-Watson test; normality was assessed using the Shapiro-Wilk test and homogeneity of variance was tested using Barlett’s test. When these assumptions were not met, non-parametric alternatives were used. In order to develop optimized protocol for each cassava variety, the effect of each MS concentration and plant growth regulators was evaluated on growth parameters using either the Kruskal-Wallis’s test or the Wilcoxson paired test. All tests were evaluated at the 5% level to determine statistical significance. Principal component analysis (PCA) and ascendant hierarchical clustering (AHC) were performed on all data to identify the most suitable media for each variety tested in the different experiments. All analyses were performed using R 4.3.1 software (Ihaka and Gentleman, 1996).

## 3 Results

### 3.1 Effect of type and concentration of cytokinin

#### 3.1.1 Effect of kinetin on *in vitro* cassava growth

**Figure 1:**
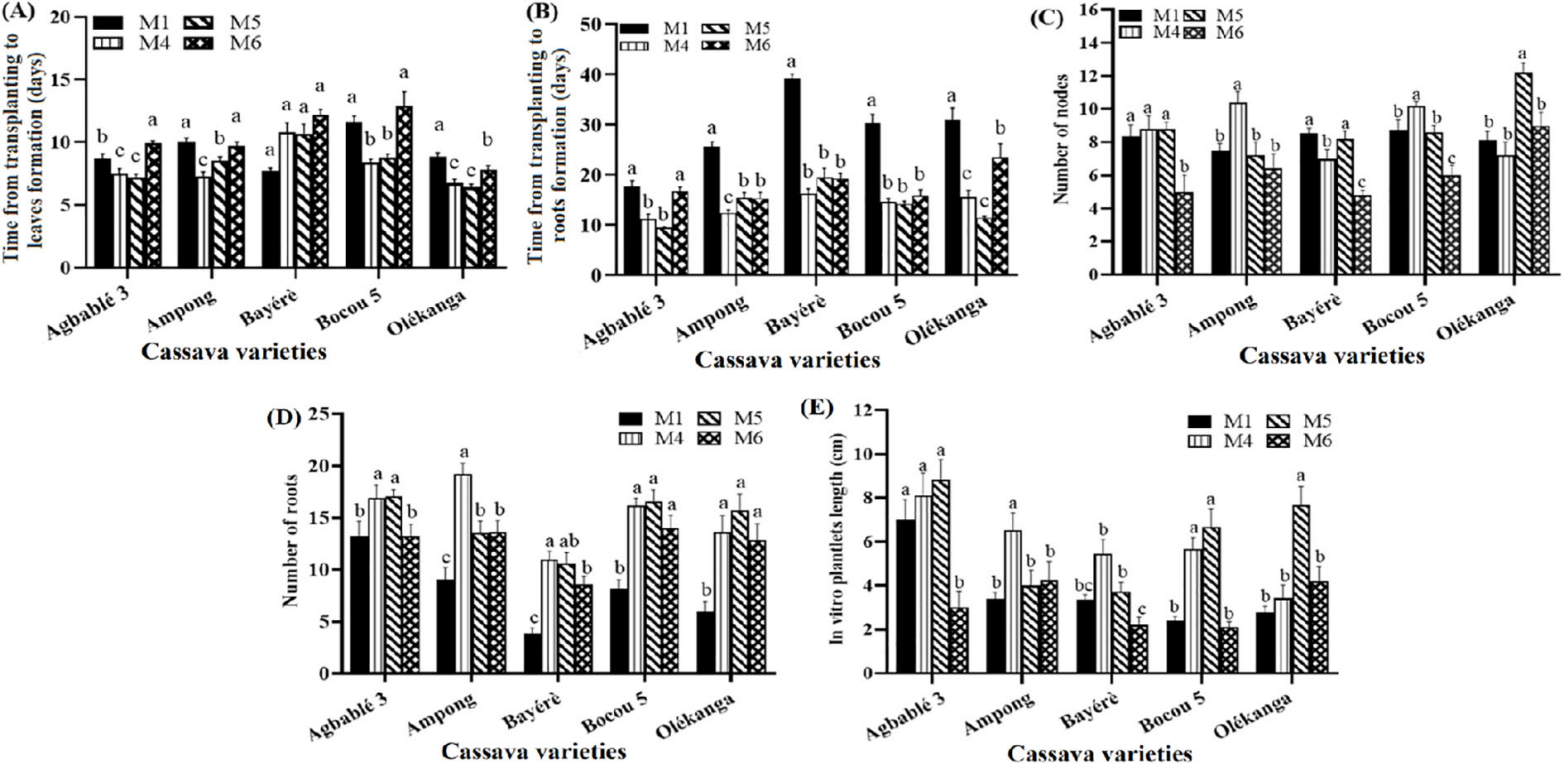
Effect of kinetin on *in vitro* organogenesis of the cassava varieties. The five cassava varieties were evaluated to determine the effect of kinetin on time from transplanting to leaves **(A)** and roots formation **(B)**, number of nodes **(C)**, number of roots **(D)** and *in vitro* plantlets length **(E)**. Data are means ± SE. The bars represent the standard error. Bars sharing the same letters are not significantly different between media for each cassava varieties (N = 30) according to non-parametric Kruskal-Wallis and pairwise Wilcoxon tests (p<0.05). M1: full-strength MS + 0.05 µM NAA + 0.22 µM BAP (control); M4: full-strength MS + 0.05 µM NAA + 0.12 µM KIN; M5: full-strength MS + 0.05 µM NAA + 0.24 µM KIN; M6: full-strength MS + 0.05 µM NAA + 0.36 µM KIN.

All the parameters evaluated differed significantly (p < 0.05) with the concentration of KIN in the full-strength MS. Kinetin at 0.12 µM (M4 medium) or 0.24 µM (M5 medium) reduced the time from transplanting to leaf emergence in three varieties, including Agbablé 3 (6-7 days on M4, M5 instead of 8-9 days on the control M1), Bocou 5 (8-9 days on M4, M5 instead of 11-12 days on M1) and Olékanga (5-6 days on M4 and 6-7 days on M5 instead of 8-9 days on M1). M4 and M5 significantly shortened this time for the Ampong variety (**Figure 1A**).

The following media induced the shortest time from transplanting to rooting for all varieties: Agbablé 3 (9-11 days on M4, M5 instead of 17-18 days on M1), Ampong (12-15 days on M4 instead of 25-26 days on M1), Bayérè (16-19 days on M4 and M6 instead of 39-40 days on M1), Bocou 5 (14-15 days on M4 and M6 instead of 29-30 days on M1) and Olékanga (11-15 days on M4, M5 instead of 30-31 days on M1) (**Figure 1B**). All the media tested induced more roots than the control medium (M1). The highest number of roots for Agbablé 3 was 17 (M5), for Ampong was 19.2 (M4), for Bayérè was 11 (M4), for Bocou 5 was 16.6 (M5) and for Olékanga was 15.7 (M5) (**Figure 1C**).

With regard to the number of nodes produced, similar results were observed for all media except one (M6: 0.36 µM KIN). In Ampong and Bocou 5, medium M4 induced a greater number of nodes than the other media, while the highest number of nodes was observed on both medium M5 and the control medium (M1) for Bayérè. Olékanga plantlets produced the highest number of nodes on the medium supplemented with 0.24 μM KIN (M5) (**Figure 1D**).

For *in vitro* plantlet length, both M4 (0.12 µM KIN) and M5 (0.24 µM KIN) gave similar means for Agbablé 3 (M5: 8.84 ± 0.5 cm and M4: 8.11 ± 1.02 cm) and Bocou 5 (M5: 6.68 ± 0.82 cm and M4: 5.68 ± 0.89 cm). M4 (0.12 µM KIN) gave the best results with Ampong (6.53 ± 0.77 cm) and Bayérè (5.46 ± 0.64 cm) plantlets, while M5 gave the highest mean plantlet length for Olékanga (7.68 ± 0.85 cm) (**Figure 1E**).

#### 3.1.2 Effect of thidiazuron on *in vitro* cassava growth

**Figure 2:**
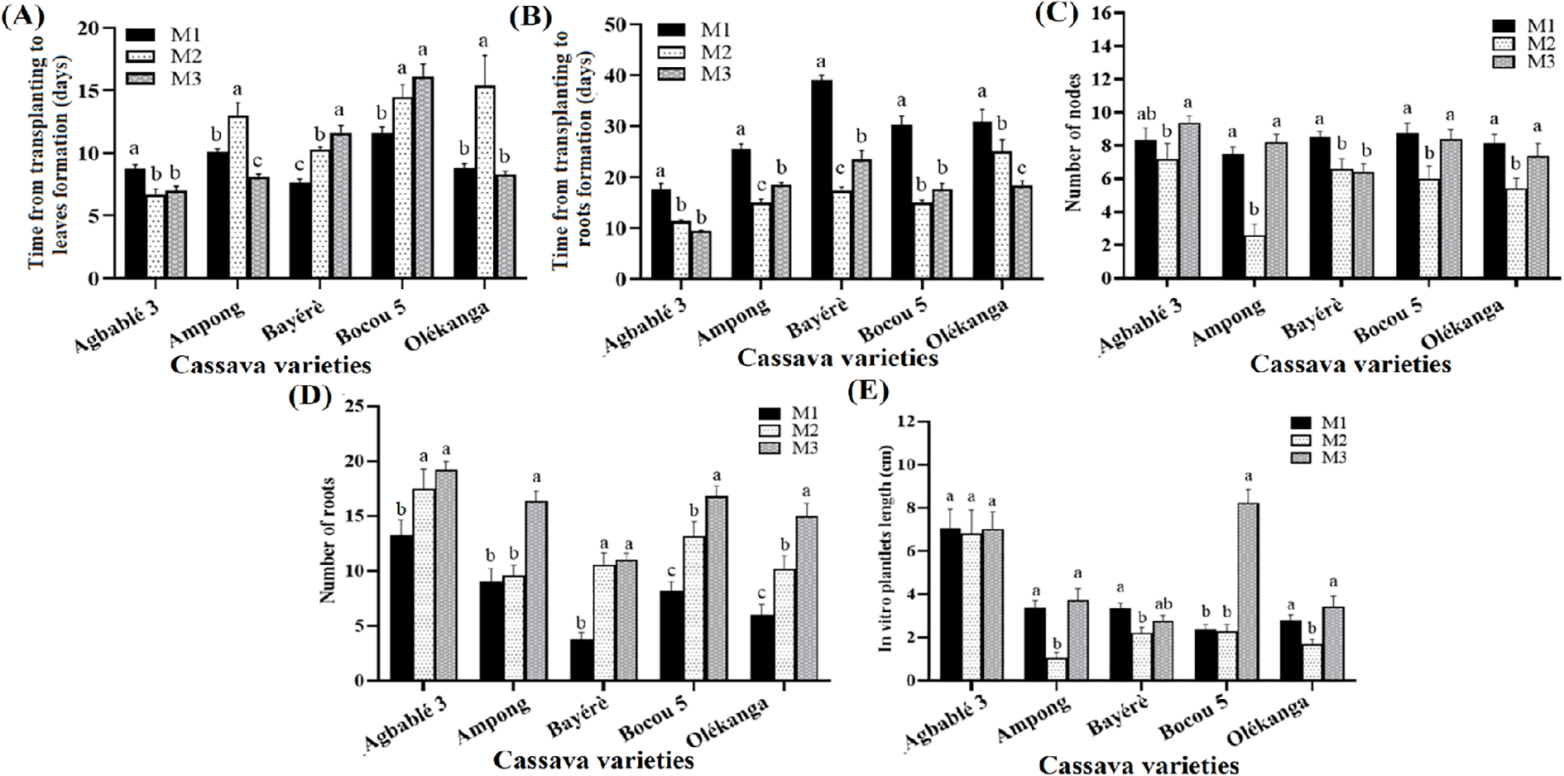
Effect of thidiazuron on *in vitro* organogenesis of the cassava varieties. The five cassava varieties were evaluated to determine the effect of thidiazuron (TDZ) on time from transplanting to leaves **(A)** and roots formation **(B)**, number of nodes **(C)**, number of roots **(D)** and *in vitro* plantlets length **(E)**. Data are means ± SE. The bars represent the standard error. Bars sharing the same letters are not significantly different between media for each cassava varieties (N = 30) according to non-parametric Kruskal-Wallis and pairwise Wilcoxon tests (p<0.05). M1: full-strength MS + 0.05 µM NAA + 0.22 µM BAP (control); M2: full-strength MS + 0.05 µM NAA + 5 nM TDZ; M3: full-strength MS + 0.05 µM NAA + 10 nM TDZ.

The addition of thidiazuron at different concentrations to the full-strength MS medium significantly affected the growth and the rooting of *in vitro* plantlets (p < 0.05). For Agbablé 3, TDZ at 5 nM (M2) and 10 nM (M3) reduced the time from transplanting to leaf production (6-7 days instead of 8-9 days in the control). Similar results were also observed for Ampong on M3 (7-8 days compared to 10-11 days in the control). In Bayérè and Bocou 5, the control medium without TDZ (M1) required less time from transplanting to leaf production (**Figure 2A**).

The time from transplanting to rooting was reduced on TDZ supplemented media for all varieties. Explants of Agbablé 3, Ampong, Bayérè, Bocou 5 and Olékanga developed roots on M2 and M3 but not on the control (M1). It was 9-11 days instead of 17-18 days for Agbablé 3; 15-18 days compared to 25-26 days in the control for Ampong; 17-23 days instead of 30-31 days for Bayérè; 15-17 days instead of 30-31 days for Bocou 5 and 18-25 days instead of 30-31 days for Olékanga explants (**Figure 2B**). Compared to the control (M1), M3 increased the number of roots for all varieties. The highest mean root numbers were 19.2, 16.2, 11.0, 16.8 and 15.0 for Agbablé 3, Ampong, Bayérè, Bocou 5 and Olékanga, respectively (**Figure 2C**).

The highest number of nodes was observed for Agbablé 3 (9.4 ± 0.41) only on M3. The node mean was high on M3 and control respectively for Ampong (8.2 and 7.5), Bocou 5 (8.4 and 8.77) and Olékanga (7.4 and 8.13). (**Figure 2D**).

The best *in vitro* plantlet length was observed on M3 for Bocou 5 (8.22 ± 0.63 cm), Ampong (3.72 ± 0.55 cm) and Olékanga (3.42 ± 0.49 cm) (**Figure 2E**).

### 3.2 Effect of MS basal salt concentration

All the parameters (time from transplanting to leaf or root formation, number of nodes, number of roots and the *in vitro* plantlet length) were significantly influenced (p < 0.05) by the different concentrations of MS basal salt used in this trial.

**Figure 3:**
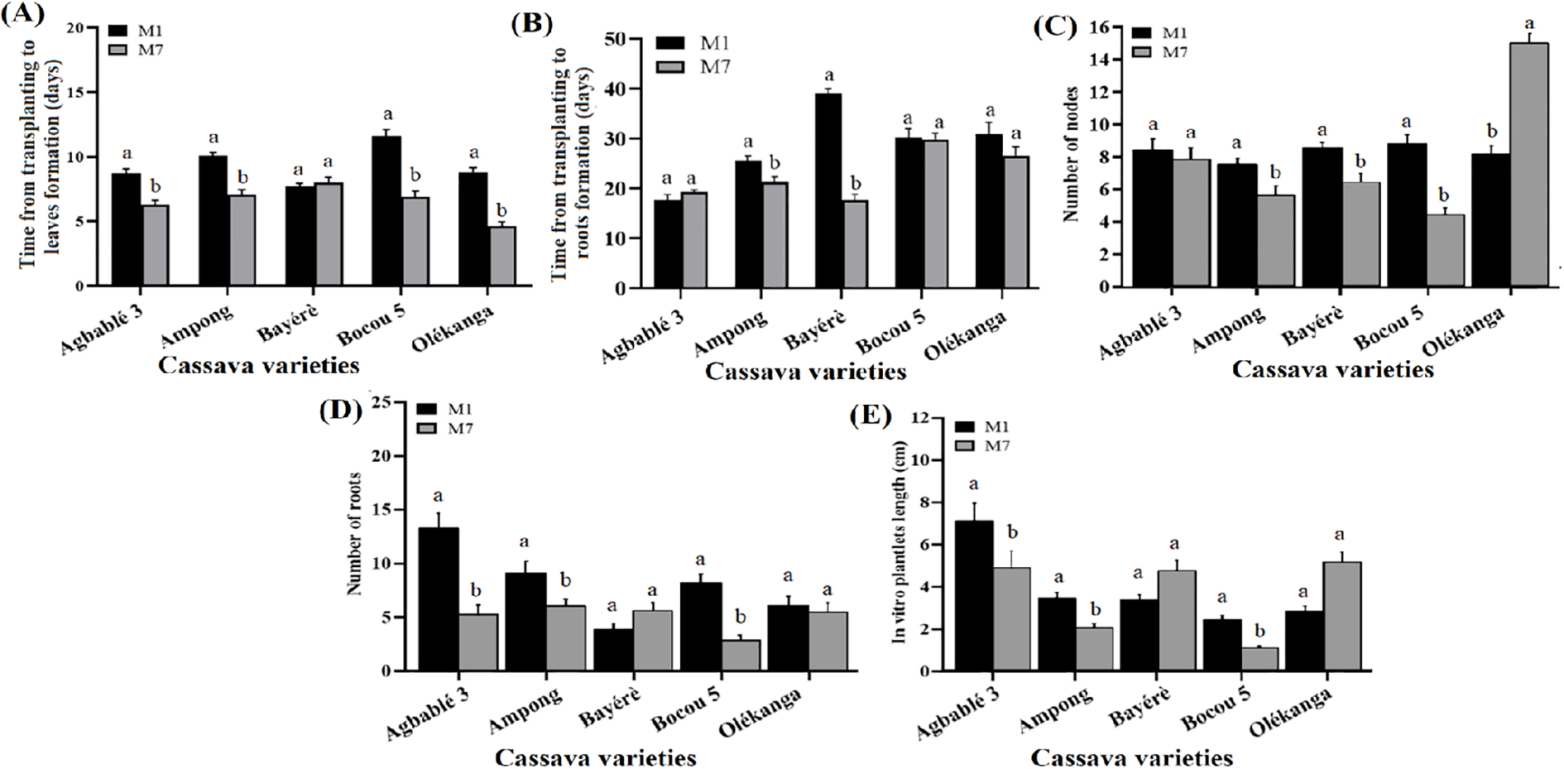
Effect of MS concentration on *in vitro* organogenesis of the cassava varieties. The five cassava varieties were evaluated to determine the effect of MS concentration on time from transplanting to leaves **(A)** and roots formation **(B)**, number of nodes **(C)**, number of roots **(D)** and *in vitro* plantlets length **(E)**. Data are means ± SE. The bars represent the standard error. Bars sharing the same letters are not significantly different between media for each cassava varieties (N = 30) according to non-parametric pairwise Wilcoxon tests (p<0.05). M1: full-strength MS + 0.05 µM NAA + 0.22 µM BAP (control); M7: half-strength MS + 0.05 µM NAA + 0.22 µM BAP.

The medium contains half-strength MS (M7) was more effective in reducing the time from transplanting to leaf formation in four varieties (**Figure 3A**), namely Agbablé 3 (6.3 ± 0.33 days instead of 8.73 ± 0.35), Ampong (7.1 ± 0.35 days instead of 10.07 ± 0.27), Bocou 5 (6.9 ± 0.44 days instead of 11.6 ± 0.51) and Olékanga (4.63 ± 0.33 instead of 8.83 ± 0.33).

The time from transplanting to rooting was also reduced by half-strength MS (M7) using in Ampong (21.3 ± 1.07 instead of 25.6 ± 0.92 days) and Bayérè (17.7 ± 1.12 instead of 39.1 ± 0.91) varieties. Nevertheless, the half-strength MS (M7) gave the same result as the full-strength MS (M1) for the other varieties (**Figure 3B**). For the number of roots, half-strength MS (M7) had induced a high mean as medium with full-strength MS (M1) for Bayérè and Olékanga; while, the highest number of roots on full-strength MS was observed for Agbablé 3 (13.27 ± 1.38 instead of 5.2 ± 0.89), Ampong (9.07 ± 1.14 instead of 6 ± 0.68) and Bocou 5 (8.17 ± 0.86 instead of 2.8 ± 0.44) varieties (**Figure 3C**).

On the other hand, full-strength MS (M1) induced the highest number of nodes in Ampong (7.5 ± 0.41 instead of 5.6 ± 0.57), Bayérè (8.53 ± 0.31 instead of 6.4 ± 0.52) and Bocou 5 (8.77 ± 0.6 instead of 4.4 ± 0.4). No significant difference was observed between the two media (M1 versus M7) for plantlets from Agbablé 3 and Olékanga (**Figure 3D**).

The highest *in vitro* plantlet length was again obtained for the varieties Agbablé 3 (7.04 ± 0.88 cm instead of 4.86 ± 0.81 cm), Ampong (3.4 ± 0.3 cm instead of 2.02 ± 0.18 cm) and Bocou 5 (2.38 ± 0.22 cm instead of 1.06 ± 0.1 cm) varieties on full-strength MS (M1) and no significant difference was observed between M1 and M7 with plantlets from Bayérè and Olékanga (**Figure 3E**).

### 3.3 Effect of the best strength of MS basal salt supplemented with the best cytokinin concentrations

**Figure 4:**
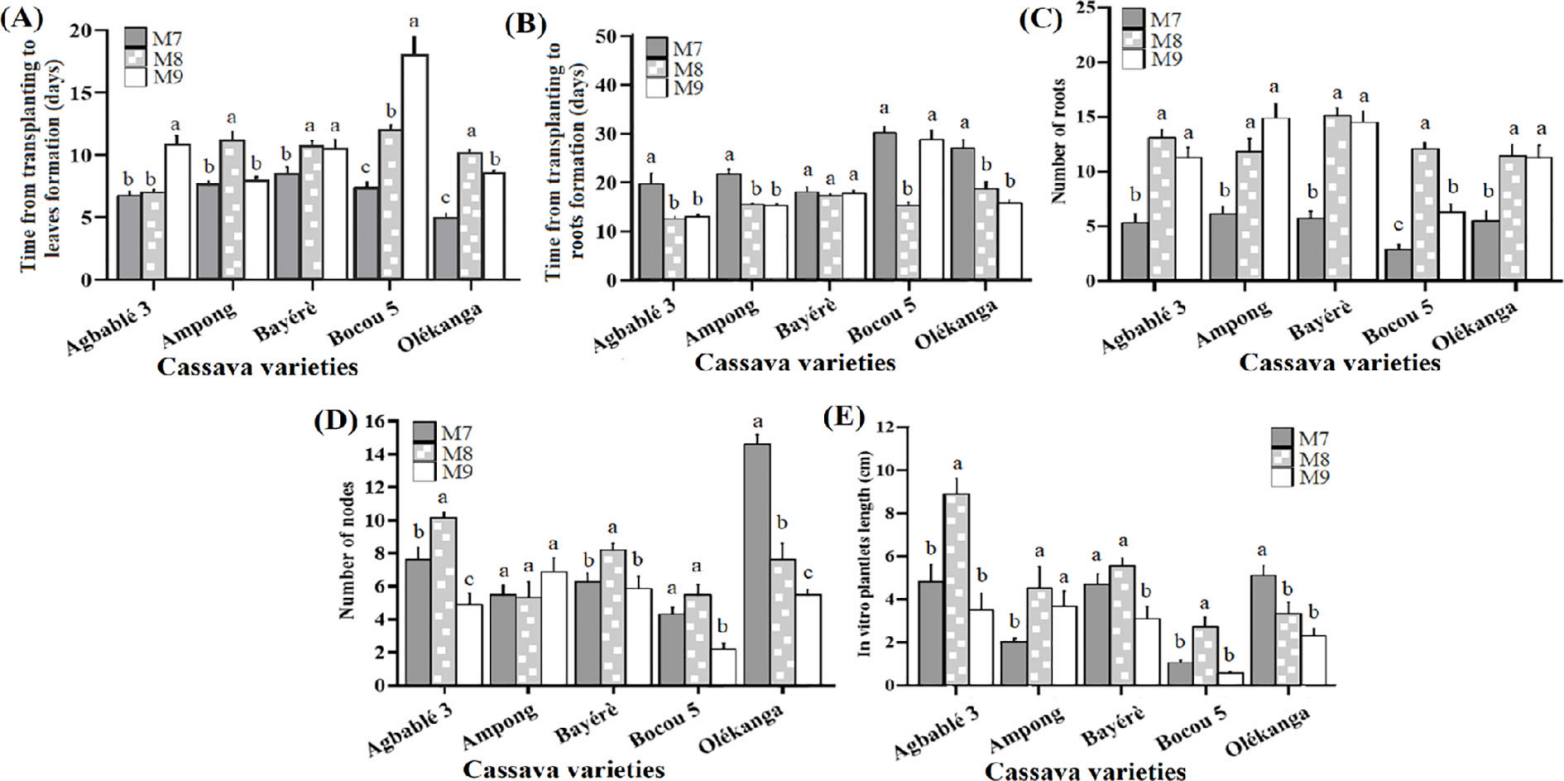
Effect of best kinetin and TDZ concentration supplemented on half-strength MS on *in vitro* organogenesis of the cassava varieties. The five cassava varieties were evaluated to determine the effect of best kinetin and TDZ concentration supplemented on half-strength MS on time from transplanting to leaves **(A)** and roots formation **(B)**, number of nodes **(C)**, number of roots **(D)** and *in vitro* plantlets length **(E)**. Data are means ± SE. The bars represent the standard error. Bars sharing the same letters are not significantly different between media for each cassava varieties (N = 30) according to non-parametric Kruskal-Wallis and pairwise Wilcoxon tests (p<0.05). M7: half-strength MS + 0.05 µM NAA + 0.22 µM BAP; M8: half-strength MS + 0.05 µM NAA + 0.24 µM KIN; M9: half-strength MS + 0.05 µM NAA + 10 nM TDZ.

From our previous results, the best strength of MS basal salt was the half-strength (M7)for reducing the time from transplanting to leaf or roots formation, while the best cytokinin concentrations were 0.24 µM KIN or 10 nM TDZ to improve organogenesis. The addition of each cytokinin concentration to half-strength MS had a significant effect (p < 0.05) on all the parameters measured. Agbablé 3 *in vitro* plantlets produced their leaves faster (between 6 and 7 days) on M8 (0.24 µM KIN) than on M7 medium supplemented with 0.22 µM of BAP (control). In Ampong, M9 (10 nM TDZ) and M7 media induced faster leaf formation. In contrast, only M7 media promoted short-term phyllogenesis with Bayérè (8-9 days), Bocou 5 (7-8 days) and Olékanga (4-5 days) explants (**Figure 4A**).

For all varieties except Bayérè, media containing KIN at 0.24 μM (M8) and TDZ at 10 nM (M9) significantly reduced the time from transplantation to root formation. In general, M8 and M9, in contrast to M7, were observed to induce roots in a short time (12-15 days) (**Figure 4B**). These media (M8 and M9) also, induced the highest number of roots for four varieties (Agbablé 3, Ampong, Bayérè and Olékanga) with the best in Bayérè on M8 (15 ± 0.77) compared to M7 (control). In Bocou 5, only M8 promoted the highest number of roots (12 ± 0.6) (**Figure 4C**).

When 0.24 μM KIN was added on half-strength MS (M8), it induced the highest number of nodes for Agbablé 3 (10.4 ± 0.36) followed by Bayérè (8.4 ± 0.4) and Bocou 5 (5.6 ± 0.63). The control medium (M7) induced the highest number of nodes (15 ± 0.6) for Olékanga plantlets (**Figures 4D**).

The highest *in vitro* plantlet length was observed on medium M8 for the varieties Agbablé 3 (8.95 ± 0.75 cm), Ampong (4.56 ± 1 cm), Bayérè (5.6 ± 0.38 cm) and Bocou 5 (2.74 ± 0.46 cm) (**Figure 4E**).

### 3.4 Identification of optimal media by principal component analysis (PCA) and ascendant hierarchical clustering

**Figure 5:**
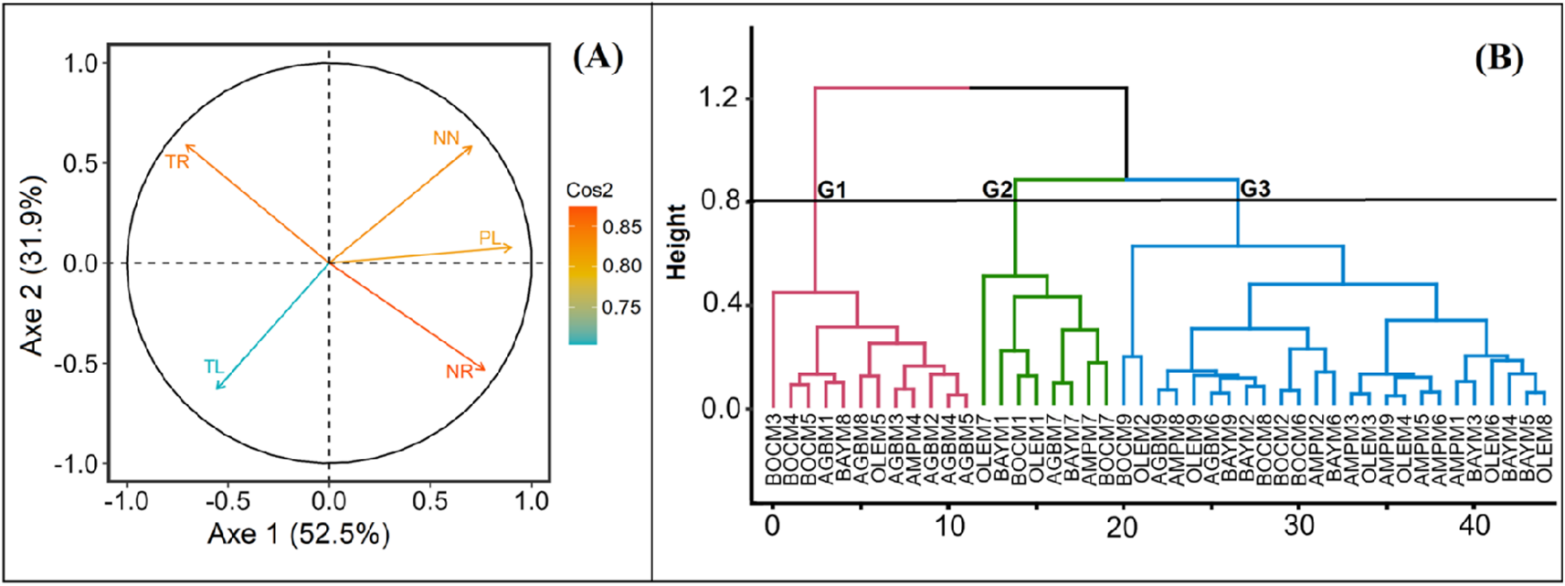
Distribution in plan formed by the axes 1 x 2. **(A)** significant variables (parameters) revealed by the correlation circle; **(B)** dendrogram of variety assigned to a medium which show the three groups; G: Group; AGB: Agbablé 3, AMP: Ampong, BAY: Bayérè, BOC: Bocou 5, OLE: Olékanga; M1: full-strength MS + 0.05 µM NAA + 0.22 µM BAP (control); M2: full-strength MS + 0.05 µM NAA + 5 nM TDZ; M3: full-strength MS + 0.05 µM NAA + 10 nM TDZ; M4: full-strength MS + 0.05 µM NAA + 0.12 µM KIN; M5: full-strength MS + 0.05 µM NAA + 0.24 µM KIN; M6: full-strength MS + 0.05 µM NAA + 0.36 µM KIN; M7: half-strength MS + 0.05 µM NAA + 0.22 µM BAP; M8: half-strength MS + 0.05 µM NAA + 0.24 µM KIN; M9: half-strength MS + 0.05 µM NAA + 10 nM TDZ. TR: Time spent from transplanting to roots production; TL: Time spent from transplanting to leaf production; NN: Number of nodes; NR: Number of roots; PL: Plantlets length; cos2: show the quality of representation.

The results of multivariate data analysis methods showed that media supplemented with some KIN and TDZ were the best media for all varieties (**Figure 5**). From the Principal Component Analysis (PCA), all the parameters evaluated were significant to describe the diversity of the media effect on the cassava varieties (**Figure 5A**). The first two axes with eigenvalues greater than 1 were retained in the analysis and explained 84.46% of the total variability observed. According to their contribution and the Ascending Hierarchical Classification (AHC), three groups were identified among the 45 treatments in all experiences.

The group I is mainly characterized by the media that induced the highest mean values of growth parameters (number of nodes: 9.2 ± 0.39; number of roots: 16.37 ± 0.56 and plantlet length: 7.27 ± 0.32 cm), except for the time from transplanting to leaf and root production for the varieties. The time from transplanting to the formation of leaves (8 days) and roots (13 days) formation was low for these media (**Table 2**). This group includes 12 treatments, mainly AGBM2: Agbablé 3 on full-strength MS + 0.05 µM NAA + 5 nM TDZ, AGBM3: Agbablé 3 on full-strength MS + 0.05 µM NAA + 10 nM TDZ, AGBM4: Agbablé 3 on full-strength MS + 0.05 µM NAA + 0.12 µM KIN, AGBM5: Agbablé 3 on full-strength MS + 0.05 µM NAA + 0.24 µM KIN, AGBM8: Agbablé 3 on half-strength MS + 0.05 µM NAA + 0.24 µM KIN, AMPM4: Ampong on full-strength MS + 0.05 µM NAA + 0.12 µM KIN, BOCM4: Bocou 5 on full-strength MS + 0.05 µM NAA + 0.12 µM KIN, BOCM5: Bocou 5 on full-strength MS + 0.05 µM NAA + 0.24 µM KIN and OLEM5: Olékanga on full-MS + 0.05 µM NAA + 0.24 µM KIN) (**Figure 5B**).

**Table 2:** Variation of the means of clusters obtained by the AHC using the five parameters.

	Growth parameters				
Cluster	Time from transplanting to roots formation (days)	Time from transplanting to leaves formation (days)	Number of nodes	Number of roots	*In vitro* plantlets length (cm)
**Group 1 (N=12)**	13.2 ± 0.86c	8.4 ± 0.77b	9.2 ± 0.39a	16.37 ± 0.56a	7.27 ± 0.32a
**Group 2 (N=8)**	26.86 ± 2.53a	7.63 ± 0.72b	8.08 ± 1.13ab	5.38 ± 0.56c	3.29 ± 0.53b
**Group 3 (N=25)**	18.11 ± 0.81b	10.56 ± 0.5a	6.22 ± 0.32b	11.95 ± 0.46b	3.07 ± 0.22b
**p-value**	**< 0.0001**	**0.0062**	**< 0.0001**	**< 0.0001**	**< 0.0001**

Comparisons were performed by Tukey tests (LSD); for each parameter per row, the values (mean ± standard error) with different letters are significantly different (p < 0.05). Bold values represent significant statistical values.

Group II includes treatments that regenerate small *in vitro* plantlets (3.07 ± 0.22 cm), with a small number of roots (5.38 ± 0.56) and less time to develop leaves (8 days) but more time (27 days) to develop roots (**Table 2**). There are AGBM7: Agbablé 3 on half-strength MS + 0.05 µM NAA + 0.22 µM BAP, BAYM1: Bayérè on full-strength MS + 0.05 µM NAA + 0.22 µM BAP (control), BAYM7: Bayérè on half-strength MS + 0.05 µM NAA + 0.22 µM BAP, BOCM1: Bocou 5 on full-strength MS + 0.05 µM NAA + 0.22 µM BAP (control), BOCM7: Bocou 5 on half-strength MS + 0.05 µM NAA + 0.22 µM BAP, OLEM1: Olékanga on full-strength MS + 0.05 µM NAA + 0.22 µM BAP (control) and OLEM7: Olékanga on half-strength MS + 0.05 µM NAA + 0.22 µM BAP (**Figure 5B**).

Group III consists of 25 treatments. The media of this group promoted shorter length (3.07 ± 0.22 cm) with small nodes (6.22 ± 0.32) and more time (11 days) to induce leaves in the cassava varieties (**Table 2**). It is characterized by AGBM9: Agbablé 3 on full-strength MS + 0.05 µM NAA + 0.36 µM KIN, AMPM8: Ampong on half-strength MS + 0.05 µM NAA + 0.24 µM KIN, BOCM1: Bocou 5 on full-strength MS + 0.05 µM NAA + 0.22 µM BAP (control), BOCM2: Bocou 5 on full-strength MS + 0.05 µM NAA + 5 nM TDZ, BOCM6: Bocou 5 on full-strength MS + 0.05 µM NAA + 0.36 µM KIN, BOCM8: Bocou 5 on half-strength MS + 0.05 µM NAA + 0.24 µM KIN (**Figure 5B** **).**

### 3.5 *In vitro* plantlets acclimatization

**Figure 6:**
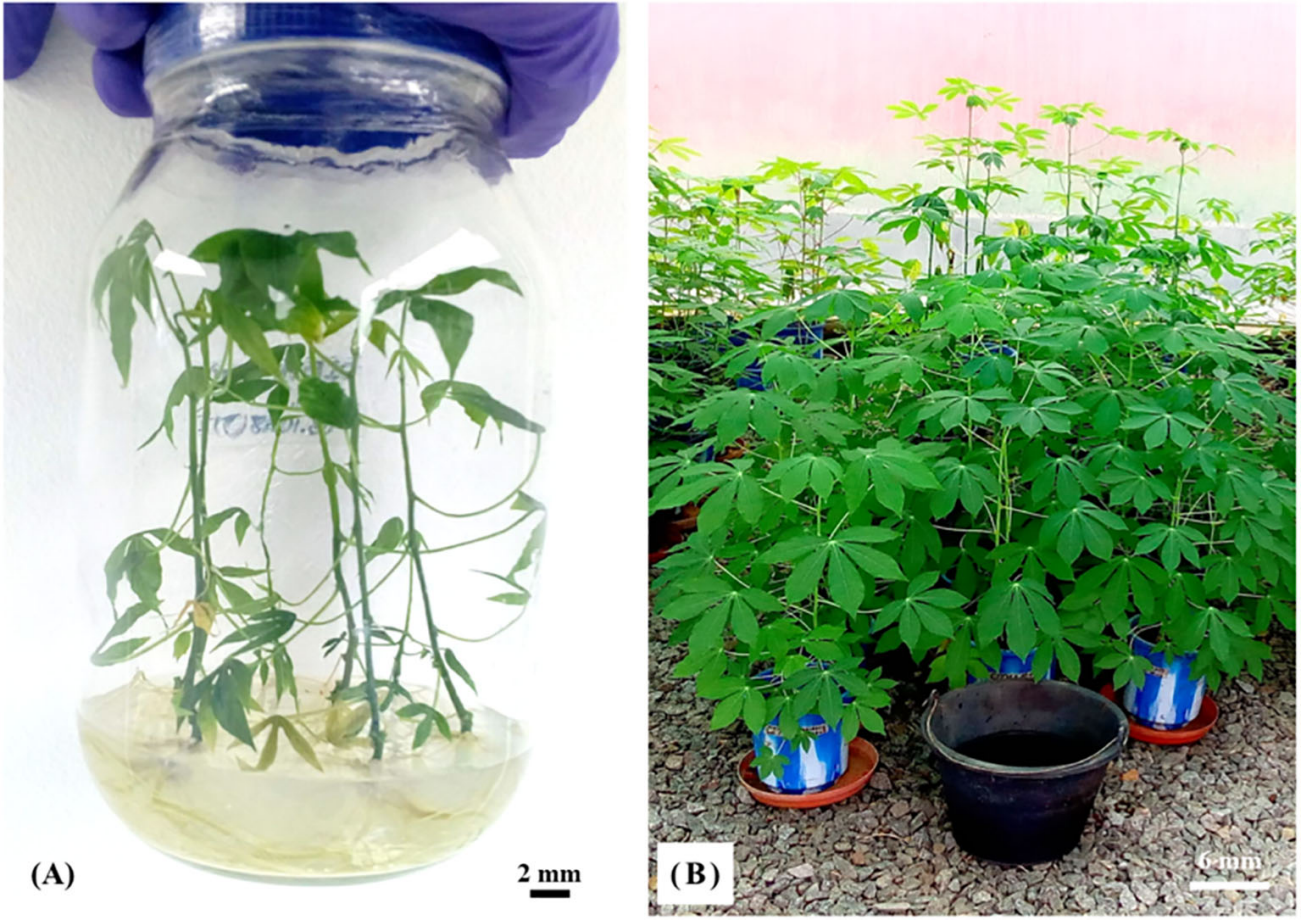
Acclimatization process of cassava varieties. *In vitro* plantlets obtained after six weeks **(A)** in the lab and hardened in the tunnel after eight weeks **(B)**.

Weaned plantlets showed good vegetative growth and no malformations (**Figure 6**). In fact, the survival rate of the weaned plants ranged from 85% to 91%. However, no significant differences were observed between all survival rates. The *in vitro* plantlets successfully adapted to *ex vitro* condition without any issues.

## 4 Discussion

To improve micropropagation, exogenous plant growth regulators (PGRs) have been introduced into the MS media to promote rapid organogenesis. The most commonly used in organogenesis are cytokinins and especially Benzylaminopurine (BAP) for cassava and often kinetin (KIN) or thidiazuron (TDZ) (Shiji et al., 2015; Sessou et al., 2020; Galán-Ávila et al., 2020; Berhanu and Feyissa, 2020). In our experience, the responses of the explants of the five cassava varieties to the different MS medium concentrations were influenced by these three PGRs (BAP, KIN, TDZ). Significant differences were observed in the times from transplanting to leaf and root formation, the number of roots, the number of nodes and the length *in vitro* plantlets. These results showed that the response of cassava to *in vitro* micropropagation depends on the culture medium components. The addition of growth regulators at different concentrations to the MS basal salt controls the *in vitro* organogenesis of this plant.

One aim of our study, was to find media that contribute to improve the number of nodes and roots. In fact, number of nodes is the most important parameter in the propagation of some species such as cassava, because the units used for propagation are made up of these organs during micropropagation (Quashie et al., 2012; Okello et al., 2021). The parameters relating to the roots are also very important, because the roots are the first to be established during weaning in order to promote the recovery of the leaves or even the plantlet. The roots must be able to convert the young plant from heterotrophic (*in vitro*) to autotrophic (*ex vitro*) life. Without a strong and hardened root system, there can be no proper weaning of the *in vitro* plantlets (Dossoukpèvi et al., 2015). So, the type, concentration and an appropriate combination of PGRs are very important to improve any regeneration system (Thorpe et al., 1991). Differentiation in plants is controlled by an interplay between auxin and cytokinin and the exogenous requirement of hormones in the medium depends on their endogenous level in the cultured plant. A high ratio of auxin to cytokinin generally results in callus formation, whereas a low ratio results in organogenesis and shoot induction. Plant organogenesis is generally regulated by endogenous auxin, i.e. the natural auxin indole acetic acid (IAA). Adding ANA, a synthetic hormone with the same properties as AIA, to the culture medium increases the auxin content of the explant. This action can create disturbances and direct the cells towards root formation. Exogenous cytokinin will regulate the formation of organs (shoots or roots) depending on the target objectives. In the current study, KIN at 0.12 µM was suitable for the multiplication of the Ampong variety while at 0.12 or 0.24 µM, it was more suitable for Agbablé 3, Bocou 5 and Olékanga. These results are similar to those of Ahanhanzo et al. (2010); Cacai et al. (2012) and Sessou et al. (2020) who showed that KIN induced better results than BAP added to the control medium for the regeneration of cassava varieties. Kinetin can therefore be used at very low concentrations for rapid micropropagation of cassava. Furthermore, some authors such as Apio et al. (2021) used higher concentrations than others and obtained different results. They found that concentrations of 2.4 ml/l (10.65 µM) of BAP followed by 2 ml/l (10.74 µM) of NAA facilitated establishment and regeneration compared to kinetin. Ahanhanzo et al. (2008) in *Dioscorea cayenensis* and Demeke et al. (2014) in cassava showed that the response of the explant varied between the genotypes they used as observed in the present study. These results effectively confirm the effect of the genotype in the *in vitro* regeneration of cassava. In addition to KIN, the results of our study also showed that thidiazuron at 5 nM or 10 nM was better in the Agbablé 3 variety. It promoted the induction of roots and leaves in a short time and contributed to the production of a high number of nodes and roots. This result could be explained by the fact that there was some interaction between the growth regulators. In other words, differentiation depended on the type of hormone combination used. These results are confirmed by the work on cassava by Berhanu and Feyissa (2020), who showed that TDZ at a concentration of 0.2 mg/l (908.1 nM) had the highest average number of shoots per explant by improving the morphology of the *in vitro* plantlets. In other work, the MS medium containing TDZ without auxins was found to have a higher number of shoots than the medium containing both TDZ and auxins (Aasim et al., 2009; Okello et al., 2021). The importance of PGRs in initiating and regulating organized development is well established. The addition of TDZ promotes shoot formation by inducing and maintaining auxin biosynthesis. This hormone regulates auxin production, promoting callus formation at high concentrations (> 5 µM TDZ), while at low concentrations (< 5 µM TDZ), shoot induction is observed (Dewir et al., 2018; Vinoth and Ravindhran, 2018). TDZ was highly effective than other cytokinins at very minimal concentrations because of its stability to withstand degradation by cytokinin oxidases (Mok et al., 1987; Pasternak and Steinmacher, 2024).

Although, the combination of PGRs with MS salt is good for propagation, Mineral elements play an important role in organogenesis. Some elements such as nitrogen (N) and potassium (K) are involved in auxin simulation. In fact, N induces IAA biosynthesis, while K increases the auxin transport system, favoring either root or shoot formation. High concentrations of mineral elements can, often, be a source of disturbance for plants. In fact, explants already contain sometime a high amount of elements or which do not have a great need for nutrients to develop (Purohit et al., 2011). Given this concern, some authors have suggested that the dilution of mineral elements in MS medium may be appropriate for some genotypes (Berhanu and Feyissa, 2020; Mazri et al., 2021). In the current study, our results showed that such dilution as half concentration of MS salt significantly reduced the time spent from transplanting to leaf formation in cassava varieties Agbablé 3, Ampong, Bocou 5 and Olékanga compared to full-strength MS which was the control. It also induced rapid rhizogenesis of Ampong and Bayérè explants. Microcuttings have some mineral elements that would be necessary for their regeneration. These microcuttings would therefore contain a high concentration of carbon, phosphorus, nitrogen, potassium, sulfur and magnesium which are macronutrients and are required in millimolar quantities. Their high concentration could, in the long term, inhibit some activities in the organogenesis process such as nitrate activity for nitrogen (Amirouche et al., 1985). In our current experiments, this was observed by the low number of nodes, roots and *in vitro* plantlet length. MS salt diluted to 50% was suitable for the rapid regeneration of the explants as shown by Mazri et al. (2021) and Quashie et al. (2012). It reduced the regeneration time. We showed also that the half-strength MS can be better for organogenesis when we add kinetin at 24µM and thidiazuron at 10 nM. The supplementary of PGRs on half strength MS is more effective in contrary when it is not supplemented. This result is due to the synergistic action of minerals and growth hormones.

Several studies have demonstrated the recalcitrant nature of some cassava varieties to *in vitro* methods. Mapayi et al. (2013) obtained significant results with one of thirteen media containing different concentrations of BAP. This medium enabled them to record the highest means for the number of leaves, nodes and heights. Similar results were observed by Sesay et al. (2018) on some recalcitrant cassava genotype. Also, Sessou et al. (2020) obtained an effective medium for each variety and these media all contained kinetin. A similar trend was observed in this study with our protocol, where the seven media containing PGRs out of the nine were all effective to overcome the recalcitrance of cassava varieties with lower concentrations for all parameters. The use of lower concentration of PGRs could contribute to reduce their very high cost, which affects the overall cost of producing material using this technique. This approach will reduce the cost of producing *in vitro* source material.

## 5 Conclusion

Our study showed that, the addition of exogenous plant growth regulators such as KIN at 0.12 and 0.24 µM and TDZ at 5 and 10 nM contributed to optimize *in vitro* micropropagation. When the concentration of Murashige & Skoog mineral salts was reduced by half, it was also effective, especially in combination with PGRs. The optimal media identified in this study also show that the response of explants of cassava varieties *in vitro* depends on the medium used and the growth parameters considered. Our protocol can therefore be used to overcome the recalcitrance of some cassava varieties that are difficult to grow using the micropropagation method. This information will be useful for mass multiplication programs and the distribution of healthy planting material to farmers.

## Data Availability

The raw data supporting the conclusions of this article will be made available by the authors, without undue reservation.

## References

[B1] Aasim M. Khawar K. M. Sancak C. Özcan S. (2009). *In vitro* shoot regeneration of Fenugreek (*Trigonella foenumgraceum* L.). American-Eurasian J. Sustain. Agric. 3, 135–138.

[B2] Ahanhanzo C. Agbangla C. Agassounon D. T. M. Cacaï G. Dramane K. (2008). Etude comparative de l’influence des régulateurs de croissance sur la morphogénèse (*in vitro*) de quelques variétés de *Manihot esculenta* Crantz (manioc-euphorbiaceae) du Bénin. Rev. CAMES-Série A 7, 47–52.

[B3] Ahanhanzo C. Gandonou C. Agbidinoukoun A. Dansi A. Agbangla C. (2010). Effect of two cytokinins in combination with acetic acid α-naphthalene on yams (*Dioscorea* spp.) genotypes’ response to *in vitro* morphogenesis. Afr. J. Biotechnol. 9, 8837–8843.

[B4] Amirouche L. Stuchbury T. Matthews S. (1985). Comparisons of cultivar performance on different nutrient media in a routine method for potato micropropagation. Potato Res. 28, 469–477. doi: 10.1007/BF02357525

[B5] Apio H. B. Alicai T. Ogwok E. (2021). Efficient conditions for *in vitro* establishment and regeneration of disease-free Ugandan farmer-preferred cassava genotypes. Afr. J. Biotechnol. 20, 369–382. doi: 10.5897/AJB2021.17361

[B6] Berhanu R. Feyissa T. (2020). Factors influencing micropropagation and somatic embryogenesis of two cassava varieties, Kello and Qulle. Cell Biol. Dev. 4 (2), 71–81. doi: 10.13057/cellbioldev/v040205

[B7] Cacai G. H. T. Ahanhanzo C. Dangou J. S. Houedjissin S. S. Agbangla C. (2012). Effets de différentes combinaisons hormonales sur l’organogenèse *in vitro* de quelques cultivars locaux et variétés améliorées de *Manihot esculenta* Crantz (manioc-Euphorbiaceae) cultivées au Bénin. Int. J. Biol. Chem. Sci. 6, 1593–1607. doi: 10.4314/ijbcs.v6i4.19

[B8] Chikoti P. C. Mulenga R. M. Tembo M. Sseruwagi P. (2019). Cassava mosaic disease: a review of a threat to cassava production in Zambia. J. Plant Pathol. 101, 467–477. doi: 10.1007/s42161-019-00255-0 31983872 PMC6951474

[B9] Costa M. C. Costa T. P. D. de Freitas Sia E. Rodrigues R. R. da Silva A. C. M. (2019). *In vitro* regeneration of shoot segments of *Manihot esculenta* varieties cultivated in northern Brazil. Plant Cell Culture Micropropagation. 15, 22–26. doi: 10.46526/pccm.2019.v15i1.138

[B10] Demeke Y. Tefera W. Dechassa N. Abebie B. (2014). Effects of plant growth regulators on *in vitro* cultured nodal explants of cassava (*Manihot esculenta* Crantz) clones. Afr. J. Biotechnol. 13 (28), 2830–2839. doi: 10.5897/AJB2013.13287

[B11] Dewir Y. H. Naidoo Y. Teixeira da Silva J. A. (2018). Thidiazuron-induced abnormalities in plant tissue cultures. Plant Cell Rep. 37, 1451–1470. doi: 10.1007/s00299-018-2326-1 30051285

[B12] Dossoukpèvi R. Dangou S. J. Cacaï G. Agbidinoukoun A. Agbangla C. Ahanhanzo C. (2015). Production et acclimatation de plants du gros basilic (*Ocimum gratissimum*) régénérés *in vitro*: effet de l’acide naphtalène acétique (ana) sur l’enracinement. Eur. Sci. J. 11 (12), 178–192.

[B13] FAOSTAT (2022). Available online at: https://www.fao.org/faostat/fr/data/QCL (Accessed November 7, 2024).

[B14] Feyisa A. S. (2021). Micropropagation of cassava (*Manihot esculenta* crantz): review. Extensive Rev. 1, 49–57. doi: 10.21467/exr.1.1.4486

[B15] Galán-Ávila A. García-Fortea E. Prohens J. Herraiz F. J. (2020). Development of a direct *in vitro* plant regeneration protocol from *Cannabis sativa* L. seedling explants: developmental morphology of shoot regeneration and ploidy level of regenerated plants. Front. Plant Sci. 11, 534467. doi: 10.3389/fpls.2020.00645 PMC7326123 32670304

[B16] Hamdeni I. Louhaichi M. Slim S. Boulila A. Bettaieb T. (2022). Incorporation of organic growth additives to enhance *in vitro* tissue culture for producing genetically stable plants. Plants 11, 3087. doi: 10.3390/plants11223087 36432813 PMC9697419

[B17] Ihaka R. Gentleman R. (1996). R: a language for data analysis and graphics. J. Comput. Graph. Stat. 5, 299–314. doi: 10.1080/10618600.1996.10474713

[B18] Kouakou B. S. M. Yoboué A. A. N. Pita J. S. Mutuku J. M. Otron D. H. Kouassi N. K.. (2024). Gradual Emergence of East African cassava mosaic Cameroon virus in Cassava Farms in Côte d’Ivoire. Agronomy 14, 418. doi: 10.3390/agronomy14030418

[B19] Mapayi E. F. Ojo D. K. Oduwaye O. A. Porbeni J. B. O. (2013). Optimization of *in vitro* propagation of cassava (*Manihot esculenta* Crantz) genotypes. J. Agric. Sci. 5, 261. doi: 10.5539/jas.v5n3p261

[B20] Mazri M. A. Bouchiha F. Anjarne M. Alfeddy M. N. Elmaataoui S. Meziani R. (2021). Effet du milieu de culture, de la source carbonée et des antioxydants sur la prolifération des bourgeons adventifs et le développement des plantules chez le palmier dattier cv. Bouskri. Afr. Mediterr. Agric. J. - Al Awamia 0, 35–49. doi: 10.34874/IMIST.PRSM/afrimed-i131.31343

[B21] Mok M. C. Mok D. W. S. Turner J. E. Mujer C. V. (1987). Biological and biochemical effects of cytokinin-active phenylurea derivatives in tissue culture systems. Hortscience 22, 1194–1197. doi: 10.21273/HORTSCI.22.6.1194

[B22] Murashige T. Skoog F. (1962). A revised medium for rapid growth and bio assays with tobacco tissue cultures. Physiologia plantarum 15, 473–497. doi: 10.1111/j.1399-3054.1962.tb08052.x

[B23] Nakabonge G. Nangonzi R. Tumwebaze B. S. Kazibwe A. Samukoya C. Baguma Y. (2020). Production of virus-free cassava through hot water therapy and two rounds of meristem tip culture. Cogent Food Agric. 6, 1800923. doi: 10.1080/23311932.2020.1800923

[B24] Ng S. Y. (1990). Culture des tissus. Le manioc en Afrique tropicale: un manuel de référence. IITA & UNICEF, 51–61.

[B25] Okello D. Yang S. Komakech R. Rahmat E. Chung Y. Gang R.. (2021). An *in vitro* propagation of *Aspilia africana* (Pers.) CD Adams, and evaluation of its anatomy and physiology of acclimatized plants. Front. Plant Sci. 12, 704896. doi: 10.3389/fpls.2021.704896 34394159 PMC8358661

[B26] Pasternak T. P. Steinmacher D. (2024). Plant growth regulation in cell and tissue culture in *vitro* . Plants 13, 327. doi: 10.3390/plants13020327 38276784 PMC10818547

[B27] Purohit S. D. Teixeira da Silva J. A. Habibi N. (2011). Current approaches for cheaper and better micropropagation technologies. Int. J. Plant Dev. Biolology 5, 1–36.

[B28] Quashie M.-L. A. Benissan A. T. Tchezoum Y. A. (2012). Micropropagation d’une plante d’intérêt nutritionnel et pharmacologique: *Moringa oleifera* Lam. J. la Recherche Scientifique l’Université Lomé 14, 7–17. doi: 10.4314/jrsul.v14i2

[B29] Rosenthal D. M. Ort D. R. (2012). Examining cassava’s potential to enhance food security under climate change. Trop. Plant Biol. 5, 30–38. doi: 10.1007/s12042-011-9086-1

[B30] Sesay J. V. Yamba N. G. G. Sherman-Kamara4 J. Quee D. D. (2018). Development of *in vitro* propagation protocol for some recalcitrant cassava (*Manihot esculenta* Crantz) genotypes in Sierra Leone. Afr. J. Biotechnol. 17, 606–613. doi: 10.5897/AJB2017.16330

[B31] Sessou A. F. Kahia J. W. Houngue J. A. Ateka E. M. Dadjo C. Ahanhanzo C. (2020). *In vitro* propagation of three mosaic disease resistant cassava cultivars. BMC Biotechnol. 20, 51. doi: 10.1186/s12896-020-00645-8 32993601 PMC7526170

[B32] Shiji R. George J. Sunitha S. Muthuraj R. (2015). Micropropagation for rapid multiplication of planting material in cassava (*Manihot esculenta* crantz). J. Root Crops 40, 23–30. Available online at: https://journal.isrc.in/index.php/jrc/article/view/229

[B33] Thorpe T. A. Harry I. S. Kumar P. P. (1991). “Application of micropropagation to forestry,” in Micropropagation: Technology and Application, eds. Debergh P. C. Zimmerman R. H. (Dordrecht: Springer Netherlands), 311–336.

[B34] Vernier P. N’Zué B. Zakhia-Rozis N. (2018). Le manioc, entre culture alimentaire et filière agro-industrielle (Editions Quae, CTA, Presses Agronomiques de Gembloux), 1–208. doi: 10.35690/978-2-7592-2708-2

[B35] Vinoth A. Ravindhran R. (2018). “ *In vitro* morphogenesis of woody plants using thidiazuron,” in Thidiazuron: from urea derivative to plant growth regulator, 211–229. doi: 10.1007/978-981-10-8004-3_10

[B36] Yéo E. F. Kouassi M. K. Pita J. S. Kouassi N. K. Koné D. N’guetta S. P. A. (2020). Using thermotherapy and meristem tip culture for producing virus-free cassava planting material from six varieties cultivated in côte d’Ivoire. Int. J. Sci. Technol. 9, 1607–1612. Available online at: https://www.ijstr.org/final-print/jan2020/Using-Thermotherapy-And-Meristem-Tip-Culture-For-Producing-Virus-free-Cassava-Planting-Material-From-Six-Varieties-Cultivated-In-Côte-Divoire.pdf

[B37] Zango A. F. V. Zinga I. Soupké R. D. L. Yandia S. P. Marabana B. T. Kassa N. G. Y.. (2021). Comparative elimination of begomoviruses in cassava meristems and axillary buds. J. Exp. Agric. Int. 43 (4), 1–9. doi: 10.9734/jeai/2021/v43i430665

